# Determination of salicylic acid content in pharmaceuticals using chitosan@Fe_3_O_4_/CPE electrode detected by SWV technique

**DOI:** 10.5599/admet.1682

**Published:** 2023-03-01

**Authors:** Sudarut Pitakrut, Phetlada Sanchayanukun, Sasithorn Muncharoen

**Affiliations:** Department of Chemistry, Faculty of Science, Burapha University, Chonburi, 20130, Thailand

**Keywords:** Salicylic acid, magnetite nanoparticles, wart treatment drugs

## Abstract

Chitosan-coated magnetite nanoparticles (Chitosan@Fe_3_O_4_) were used to modify the carbon paste electrode (Chitosan@Fe_3_O_4_/CPE) to enhance sensitivity for salicylic acid (SA) analysis using square wave voltammetry (SWV). The performance and behaviour of the purposed electrodes were investigated using cyclic voltammetry (CV). The results showed that the mixed behaviour process was observed. Furthermore, parameters affecting SWV were also studied. It was discovered that the optimum conditions were a two-linearity range of SA determination, 1-100 and 100-400 μM. The limit of detection (LOD) and the limit of quantitation (LOQ) for SA are 0.57 μM and 0.90 μM, respectively. The proposed electrodes were successfully used to determine SA in applications employing pharmaceutical samples.

## Introduction

2-hydroxybenzoic acid, also known as salicylic acid (SA) [1], has played a very important role in daily human life from the past to the present. Due to its antibacterial properties, SA has been used in many fields, such as in the food industries and agriculture. SA is used to control plant diseases and prolong the spoilage of agricultural products. It is also used as a preservative in fermented food [2]. Additionally, in cosmetic and pharmaceutical industries, SA is an ingredient in creams or serums for treating acne or wart, including various skin medicines because of its antiseptic and anti-inflammatory properties [3,4]. Although SA is enormously useful, it is also dangerous for humans. Given high doses of SA, fever, difficulty breathing, cancer, tinnitus and possibly death are detected [2].

Consequently, the analysis of SA content has attracted great interest from scientists. The literature has shown that there are several popular techniques for SA analysis, such as high-performance liquid chromatography, UV-visible spectrophotometry and fluorescence spectroscopy [5-7]. Nonetheless, the above-mentioned techniques, even with high accuracy and precision, have limitations on the size of the tool, high cost and require experts to analyse. One of the most popular techniques used for SA analysis is various modes of electrochemical techniques. They are quick techniques with the potential to fabricate a small-size device. The improvement of these analytical electrochemical techniques is mostly a modification of the working electrodes to improve sensitivity and selectivity.

Currently, nanoparticles have received a lot of attention for their good catalytic properties. These can conduct electricity and improve the surface of the working electrode, causing to increase the analytical efficiency even higher [8,9]. One of the nanoparticles that are very popularly used in analytical electrochemical techniques is magnetite nanoparticles (Fe_3_O_4_) [10,13]. Particularly, the magnetite nanoparticles were combined with materials such as gold nanoparticles, carbon nanotubes and reduced graphene oxide, including chitosan [14-16]. The surface of the magnetite nanoparticles was enhanced with chitosan to enhance the analytical efficiency due to adsorption between positively charged chitosan and negatively charged species [17], like SA in this work. Hence, the notable aim of this work is to use the magnetite nanoparticles coated with chitosan (Chitosan@Fe_3_O_4_/CPE) as the working electrode to enhance the analytical efficiency [19] for SA determination in wart treatment pharmaceuticals.

## Experimental

### Reagents and materials

Distilled water (DI water) was used throughout all experiments from a Milli-Q Plus System (Millipore). All chemicals used in this work were analytical-grade reagents (AR-grade). Salicylic acid (C_7_H_6_O_3_) was purchased from Rankem (India). Potassium dihydrogen phosphate (KH_2_PO_4_), acetone (C_3_H_6_O) and 99 % ethanol (C_2_H_5_OH) from QRËC™ (New Zealand) were obtained. Graphite powder (≤20 μm powder) and Whatman™ qualitative filter paper, grade 1 from Sigma-Aldrich (Island) and paraffin oil from Fisher Scientific (USA) were used for the preparation of the proposed electrode.

### Fabrication of the electrode

The fabrication procedure of the carbon paste electrode modified with chitosan@Fe_3_O_4_ in this work was adopted from [18]. Briefly, it was prepared by weighing 0.0800 g of the graphite powder and 0.0100 g of chitosan@Fe_3_O_4_. Then 1.0 mL acetone was added into the mixture and sonicated using an ultrasonic homogenizer (ZEALWAY/China) for 30 min. It became the carbon paste modified with chitosan@Fe_3_O_4_. After that, 50 μL paraffin oil was added to the paste and mixed up. The paste was compressed into an electrode mole (BAS/Japan) until tight. The carbon paste electrode modified with chitosan@Fe_3_O_4_ was polished using WhatmanTM qualitative filter paper grade 1 to smooth the electrode surface.

### Apparatus

The potentiostat (Autolab PGSTAT204, Metrohm, Netherlands) were used for all electrochemical experiment. A conventional three-electrode system: chitosan@Fe3O4 modified CPE (chitosan@Fe_3_O_4_/CPE), Ag/AgCl (in 3 M KCl), platinum wire as a working electrode, a reference electrode and an auxiliary electrode were utilized. For the standard method, a specord 210 plus UV-Visible spectrophotometer (Analytica Jana/ Germany) was used.

### Electrochemical methods

The square wave voltammetry (SWV) was carried out in a 0.1 M phosphate buffer solution containing pH 5. Scan potential 0.2 to 1.4 V, frequency 50 Hz, amplitude 50 mV/s, step potential 10 mV/s, and pre-concentration step at potential 0.2 V for 30 s were used as voltammetric parameters. The results of the study of the optimum conditions for SWV parameters are shown in Figure S1 in the Supplementary document. The electrochemical impedance spectra (EIS) were created using a frequency range of 0.1 Hz to 10 kHz and an amplitude of 10 mV/s at a voltage of 0.2 V.

### Sample analysis

Generally, SA is one of the important ingredients in medicine for treating warts. Then, wart medicine was selected as a sample in this study. These samples are usually paste-like. Thus, all these experiments were treated the same way as in SA solutions. In this work, the standard addition was selected to determine SA in all samples. Briefly, the samples were diluted 200 times with 70 %v/v ethanol to determine the amount of SA using the square wave voltammetry technique.

## Results and discussion

### Electrochemical response of SA on chitosan@Fe_3_O_4_/CPE

**Figure 1.**
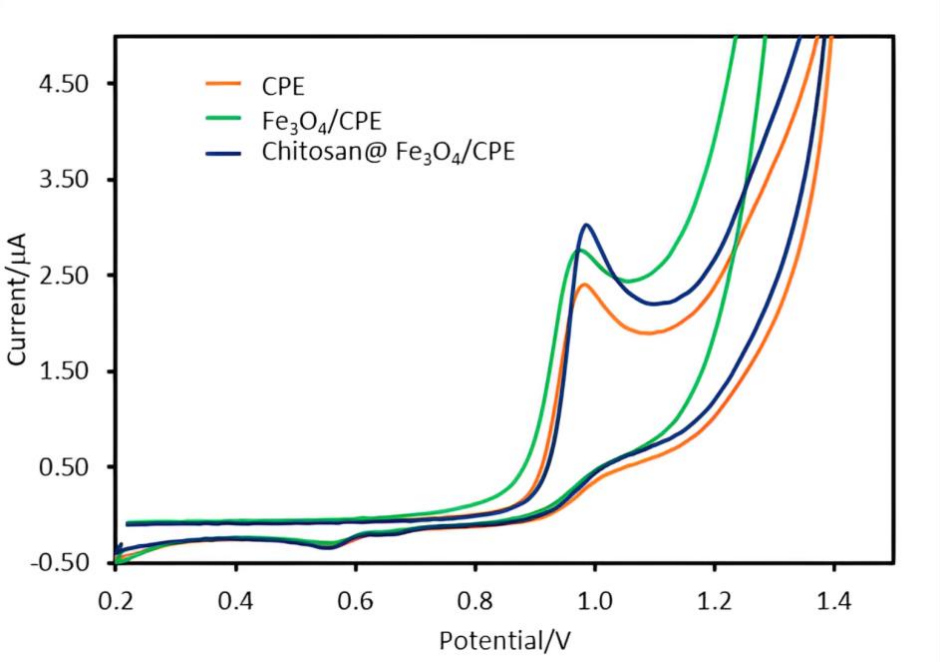
Cyclic voltammograms of 50 μM salicylic acid (SA) in 0.1 M phosphate buffer pH 5, scan rate 100 mV/s on bared CPE (orange line), modified carbon paste electrode (Fe_3_O_4_/CPE) (green line) and chitosan coated on Fe_3_O_4_ modified CPE (chitosan@Fe_3_O_4_/CPE) (blue line)

The responses of SA on various electrodes: bare CPE, Fe_3_O_4_ modified carbon paste electrode (Fe_3_O_4_/CPE) and chitosan-coated on Fe_3_O_4_ modified CPE (chitosan@Fe_3_O_4_/CPE) were studied. The results demonstrate that the oxidation current of SA detected by chitosan@Fe_3_O_4_/CPE (I = 3.0 μA) gave higher currents than on the bare CPE (I = 2.4 μA) and Fe_3_O_4_/CPE (I = 2.7 μA) as shown in Figure 1. It was observed that the nanoparticles-modified CPE showed higher currents compared to bared CPE due to the high surface area from the nanoparticles [10]. The surface area of the modified (chitosan@Fe_3_O_4_/CPE) and unmodified electrodes (bare CPE) was calculated according to the Randles-Ševčík equation [20]. It was found that the electroactive surface area of 30.11 mm^2^ and 24.09 mm^2^ for chitosan@Fe_3_O_4_/CPE and bared CPE, respectively. Additionally, another reason for the higher observed current from chitosan@Fe_3_O_4_/CPE may be the attraction between the protonated amine group of chitosan coating on the Fe_3_O_4_ and SA, which leads to the enhancement of response currents according to our previous work [18]. The proposed mechanism between SA and electrode surface is shown in Figure S2 (supplementary).

**Figure 2.**
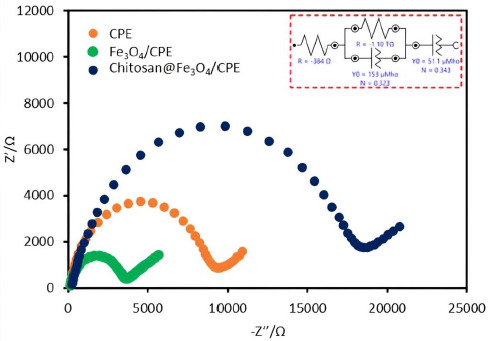
Nyquist diagrams of bared CPE (orange line), Fe_3_O_4_/CPE (green line) and chitosan@ Fe_3_O_4_/CPE (blue line), and equivalent circuit model of the chitosan@Fe_3_O_4_/CPE (inset).

Additionally, the electrochemical impedance spectroscopy (EIS) was used for the investigation of the proposed electrode surface. EIS data can be represented by a Nyquist diagram from which some conclusions about the interface properties of the electrode can be drawn. Generally, the frequency dependence of EIS can be divided into two regions, semi-circular and straight-line. The semi-circular sections show the resistances of the electrodes. In the case of a narrow semicircle, the resistance is small or there is good electrical conductivity. On the other hand, a wide semicircle indicates a large electrical resistance or lower conductivity. The straight-line section depicted the diffusion process of the electrodes [21,22]. EIS data of 0.5 mM Fe(II) in 0.1 M KNO_3_ were obtained for three various types of electrodes: bare CPE (orange line), Fe_3_O_4_/CPE (green line) and chitosan@Fe_3_O_4_/CPE (blue line) (Figure 2). From the results, it can be observed that the smallest semicircle was obtained for Fe_3_O_4_/CPE electrode. This is because of the good electrical conductivity and fast electron transfer rates at Fe_3_O_4_ nanoparticles. Although the chitosan@Fe_3_O_4_/CPE similarly contains Fe_3_O_4_ nanoparticles, the surface of these particles is encapsulated with chitosan. Therefore, the chitosan@Fe_3_O_4_/CPE shows the wider semicircle part in Nyquist diagrams compared to the Fe_3_O_4_/CPE. However the oxidation current of SA detected with chitosan@Fe_3_O_4_/CPE illustrated the highest response (Figure 1), which may help confirm the attraction between the protonated amine group of chitosan favours the electrochemical reaction.

### Electrochemical behaviour

**Figure 3.**
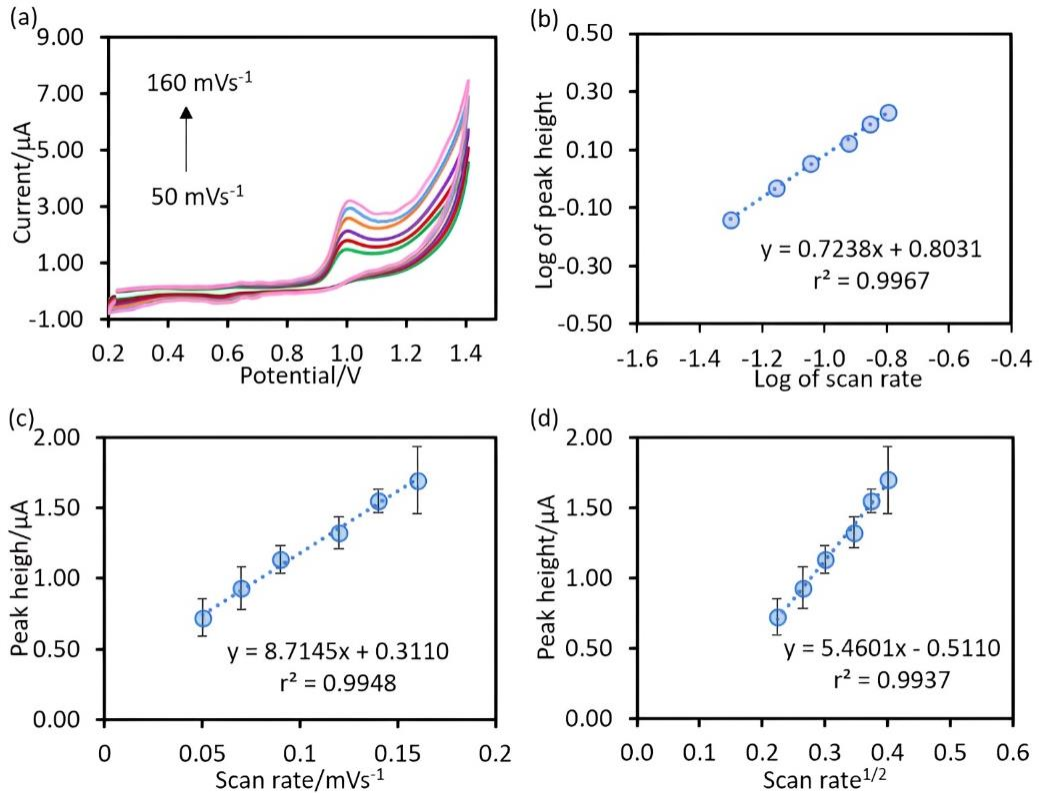
(**a**) Cyclic voltammogram of 50 μM SA in 0.1 M phosphate buffer pH 5 at different scan rates (50-160 mV/s), (**b**) plot between of logarithm of peak current and the logarithm of scan rate, (**c**) plot between of the peak current and the scan rate and (**d**) plot between of peak current and square root of scan rate for 50 μM salicylic acid in 0.1 M phosphate buffer pH 5

The behaviour of the chitosan@Fe_3_O_4_/CPE as the working electrode was studied using cyclic voltammetry at various scan rates (50-160 mV/s) (Figure 3(a)). The results shown in Figure 3 were plotted as follows: Figure 3(b) is the plot between the logarithm of peak current (y-axis) and logarithm of scan rate (x-axis) to study the mixed behaviour (adsorption and diffusion), Figure 3(c) is the plot between the peak current (y-axis) and the scan rate (x-axis) to study adsorption process and Figure 3(d) is the plot between the peak current (y-axis) and square root of scan rate (x-axis) to study diffusion process [23]. As considering the slope value of the plot in Figure 3(b), this value (0.7238) was greater than 0.5 but less than 1.0, indicating that charge transfer is under mixed control according to Wyantuti, Hartati, Panatarani & Tjokronegoro [24]. Additionally, to confirm the behaviour of the electrode, the relative coefficients of Figure 3(c) and Figure 3(d) were compared. It was found that the relative coefficients of both Figure 3(c) and Figure 3(d) were close to 1, indicating that the electron transfer at the developed electrodes is under mixed control, which is consistent with the above [24].

### Effect of pH

**Figure 4.**
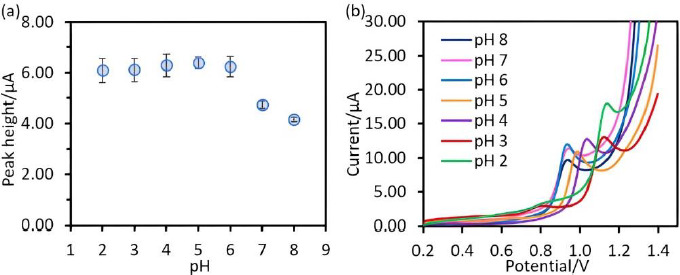
(**a**) A plot between of peak current and various pH (**b**) the square wave voltammograms of salicylic acid 50 μM in 0.1 M phosphate buffer. The measurement conditions: deposition time 30 s, deposition potential 0.2 V, frequency 50 Hz, amplitude 50 mV/s, step potential 10 mV/s.

In this work, the study of the electrochemical response of SA in dependence on the solution pH was done in a phosphate buffer in the pH range between 2 to 8 because of p*K*a1 of SA is 2.9 [25]. The results show that the oxidation current signals of SA detected by Chitosan@Fe_3_O_4_/CPE in the pH range between 2-6 exhibit no differrence, while the signals decreased at pH values above 7, as shown in Figure 4(a). Due to the deprotonated amine group of chitosan (pH 7-8), the attraction between chitosan and SA was diminished. However, in a highly acidic medium, losing of the cross-linkage between chitosan and glutaraldehyde occurred, as referred to in the report of Freire *et al.* [11]. Additionally, it was found that SA responses at pH 2-4 were slightly lower than the SA response at pH 5 as shown in Figure 4(b). Therefore, pH 5 was selected as the optimum medium for further work.

### Interference study

Electrochemical techniques can detect the following sample interferences: resorcinol, phenol, lactic acid, and fluorouracil, each to a different extent. In this study, the measurements were conducted by adding interference to 50 μM SA under optimum conditions. As shown in Table 1, the tolerance limit is set to have a relative error of not more than 10 % [26]. In addition, for sample analysis, the standard addition method was used, the determination should not also be affected by the interfering agents.

### Analytical performance

#### Linear range

**Figure 5.**
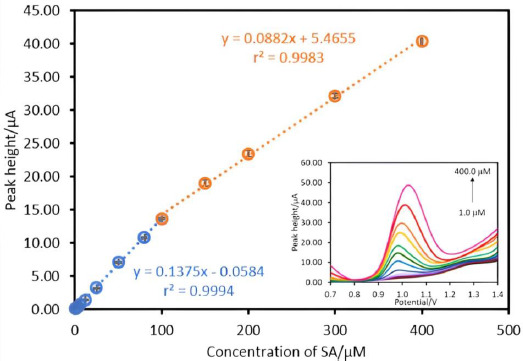
The calibration plots of SA detected by Chitosan@Fe_3_O_4_/CPE, insert the square wave voltammograms of salicylic acid 1.0 to 100 μM and 100.0 to 400.0 μM in 0.1 M phosphate buffer pH 5. The measurement conditions: deposition time 30 s, deposition potential 0.2 V, frequency 50 Hz, amplitude 50 mV/s, step potential 10 mV/s.

The various concentrations of SA (1.0-400.0 μM) were measured by the proposed electrode under optimal conditions using square wave stripping voltammetry (SWV). The calibration plots between the various concentrations of SA and oxidation peak current are indicated in Figure 5. It was found that the linearity was in the range of 1.0-100.0 μM (r^2^=0.9994) and 100.0-400.0 μM (r^2^=0.9983).

#### Limit of detection (LOD) and limit of quantitation (LOQ)

The detection limit can be calculated from 3s.d./slope and the limit of quantitation is calculated from 10SD/slope in which s.d. is the standard deviation and slope from the calibration plot. The measured detection and the quantitation limits were 0.57 and 0.90 μM, respectively, as shown in Table 2.

#### Repeatability

The stability of the proposed electrode was investigated by measurement of 50 μM SA in 0.1 M phosphate buffer pH 5 using the same electrode. A relative standard deviation (%RSD) was 1.17 % (n=35), according to the AOAC standard stating that %RSD has not to exceed 7.3 % (Table 2) [27].

### Application for real samples

As mentioned above, the standard addition was used for SA analysis in all samples. The obtained results were compared with those of UV-Visible spectrophotometry as the standard method, as shown in Table 3. It was found that the two methods were not significantly different at the 95% confidence level (*t*_stat_ = 0.69, *t*_crit_ = 2.78). In addition, the recovery percentages of samples adding with 3 concentrations of 10.00-30.00 mM SA were in the range of 94.40-113.51 that remained within the acceptable range (85-115 %) in response to the AOAC standard (Table 4) [27].

### Comparison with previously published research

The comparison of the proposed method using Chitosan@Fe_3_O_4_/CPE with some studies in the literature is shown in Table 5. The procedure of all reports shown in this table consisted of various voltammetric modes and different working electrode types. It was discovered that most of the electrodes modified with nanoparticles showed high sensitivity, including this work [2,4,28-31]. Although techniques for electrode preparation using nanoparticles require complicated electrode fabrication methods, a suitable wide working range for determining SA in the pharmaceutical samples is found especially in this work.

## Conclusions

In this work, the carbon paste electrodes modified with chitosan-coated magnetite nanoparticles (Chitosan@Fe_3_O_4_/CPE) were developed for SA analysis. At acidic conditions, the amine proton of chitosan at magnetite nanoparticle as the positively charged surface attracted the negatively charged SA increasing the sensitivity and selectivity of SA analysis. Under the optimum conditions, the linearity ranges of 1.0-100.0 and 100.0-400.0 μM with r^2^ of 0.9994 and 0.9983) were observed. The proposed method gave high sensitivity (LOD = 0.57 μM). The steadiness of the proposed electrodes as precision showed actual stability with 1.17 %RSD (n = 35). Furthermore, this method was also successfully applied for the determination of SA in wart treatment pharmaceutical samples.



## Figures and Tables

**Table 1.** Interferences study on chitosan@Fe_3_O_4_/CPE for determination of SA in drug to treat warts sample.

Interference agent	Concentration ratio of SA: IF	Tolerance limit (%RSD)
Resorcinol	1:0.5	5.5
Phenol	1:0.1	6.6
Lactic acid	1:1000	3.8
Fluorouracil	1:2	9.2

*IF : Interference agent, SA: salicylic acid

**Table 2.** Analytical performance for the determination of SA under optimal conditions

Parameter	Result
Linear range (μM)	1.0-100.0 and 100.0-400.0
Linear equation	
1.0-100.0 μM	y = 0.1375x - 0.0584 (r^2^ = 0.9994)
100.0-400.0 μM	y = 0.0882x + 5.4656 (r^2^ = 0.9983)
Limit of detection (LOD) (μM)	0.57
Limit of quantification (LOQ) (μM)	0.90
% RSD (n = 35)	1.17

**Table 3.** Comparison of SA content analysis in pharmaceutical samples using between the standard method (UV-Visible spectrophotometric method) and the proposed method.

Sample	Proposed method (mM) (n=3)	Standard method (mM) (n=3)
S1	1.39±0.04	1.39±0.00
S2	0.84±0.08	0.86±0.00
S3	0.60±0.03	0.62±0.00
S4	0.93±0.04	0.91±0.00
S5	0.06±0.02	0.06±0.00

**Table 4.** Recovery percentage for determination of SA in samples using the developed method

Sample	Concentration of SA, μM		Recovery, % (n = 3)
	Add	Found (mean ± SD; n = 3)	
S1	0.00	13.94 ± 0.38	-
	10.00	23.47 ± 0.79	95.32
	20.00	34.22 ± 1.36	101.39
	30.00	43.77 ± 2.00	99.42
S2	0.00	10.08 ± 0.93	-
	10.00	21.12 ± 1.53	110.61
	20.00	31.32 ± 3.23	106.33
	30.00	43.77 ± 2.00	112.36
S3	0.00	7.07 + 0.49	-
	10.00	17.19 ± 1.19	98.64
	20.00	28.79 ± 1.70	107.15
	30.00	41.41 ± 2.89	113.51
S4	0.00	7.07 ± 0.49	-
	10.00	18.06 ± 1.20	110.06
	20.00	28.89 ± 2.13	106.95
	30.00	39.81 ± 3.91	107.67
S5	0.00	9.91 ± 0.12	-
	10.00	19.53 ± 0.07	94.40
	20.00	30.54 ± 0.56	103.14
	30.00	41.13 ± 3.21	104.08

**Table 5.** A comparison of the performance of the proposed electrodes with previous studies to determine SA.

Electrode	Technique	Sample	Linear range, μM	LOD, μM	Ref.
NiTiO_3_/CPE	DPV	Aloe vera	3-40 and 40-1000	0.068	[28]
MIP/GCE	SWV	-	60-100	20	[29]
SPCE	DPV	Pickled vegetables and fruits juice	1-200	1.6	[2]
SPCE	SWV	Urine	16-300	5.6	[30]
Ce/ZrO_2_/CPE	SWV	human serum, milk and pharmaceuticals	5-1000	1.1	[4]
GCE	DPV	pharmaceuticals	7-434	0.65	[31]
Chitosan@Fe_3_O_4_/CPE	SWV	pharmaceuticals	1-100 and 100-400	0.57	this work
