# Supplementary material for: Determination of salicylic acid content in pharmaceuticals using chitosan@Fe3O4/CPE electrode detected by SWV technique
Source: ADMET DMPK. 2023 Mar 1;11(2):175–84. doi: 10.5599/admet.1682 (PMC10262223; doi:10.5599/admet.1682)
Supplement: Supplementary file 1 [file ADMET-11-1682-S1.pdf]

ADMET & DMPK 11(2) (2023) S1-S2

Open Access : ISSN : 1848-7718

http://www.pub.iapchem.org/ojs/index.php/admet/index

Supplementary material to

Determination of salicylic acid content in pharmaceuticals using chitosan@Fe_3O_4/CPE electrode detected by SWV technique

Sudarut Pitakrut, Phetlada Sanchayanukun and Sasithorn Muncharoen*

Department of Chemistry, Faculty of Science, Burapha University, Chonburi, 20130, Thailand

ADMET & DMPK 11(2) (2023) 175-184; doi: https://doi.org/10.5599/admet.1682

Study of optimum SWV parameters

The optimum conditions for SWV parameters, for instance deposition time, frequency, pulse amplitude and step potential were studied in this work.

For deposition time study, it is an important step known as the preconcentration step in stripping voltametric technique. For this study, the deposition potential controlled at 0.2 V to make sure that no SA oxidation was investigated. The deposition time can affect the shape and observed signal height. As shown in Figure S1(a), the peak height increased from 0 to 30 s and slightly decreased from 30 to 120 s due to electrode surface saturation. Thus, the deposition potential and time at 0.2 V and 30 s, respectively, were selected for further study.

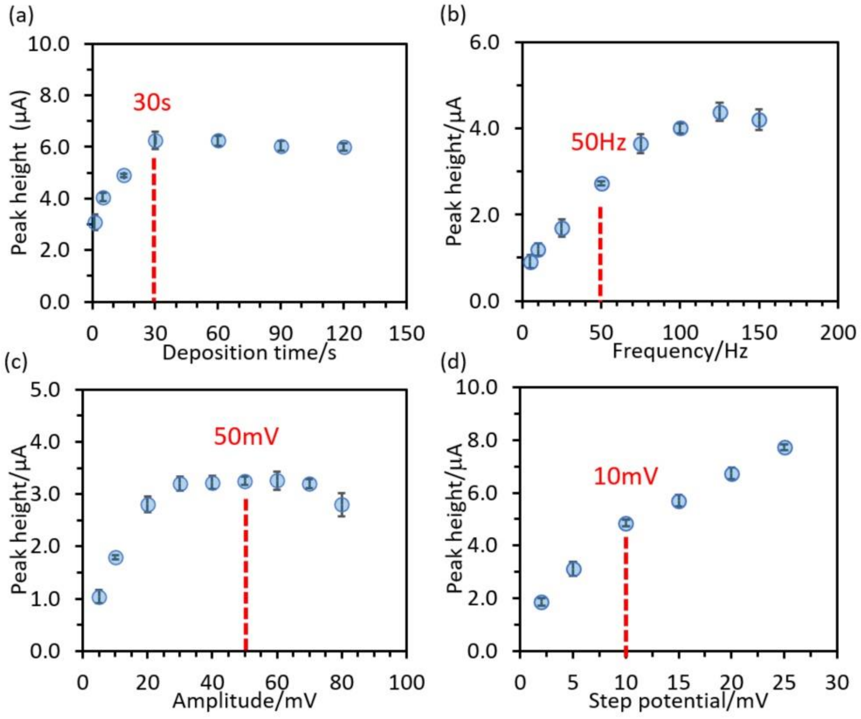

Figure S1. The relationship between peak height and parameter of square wave voltammetry (a) deposition time, (b) frequency, (c) pulse amplitude and (d) step potential of SA analysis in 0.1 M PBS pH 5.

For frequency study, it was observed that varying frequency from 5 to 150 Hz the frequency was more increased, the signals more enhanced as shown in Figure S1(b). Therefore, frequency of 50 Hz was selected because of suitable current and standard deviation.

For pulse amplitude study, the pulse amplitudes varied from 2 to 80 mV. As the results, the peak current was increased, when the amplitude increased by varying 5 – 30 mV (Figure S1(c)). The amplitude varied from 30 to 70 mV s^{-1} gave constant current. The amplitude at 50 mV was chosen as optimum pulse amplitude for the analysis.

For step potential study, varying from 2 to 25 mV of step potentials was studied. The results showed that the more increase potential values applied, the more enhance signals been gotten. Thus, 10 mV step potential was selected because it was showing the appropriate current and small standard deviation including symmetric signal. The SWV voltammograms at the optimum conditions for SA analysis were shown in Figure 5 (inset).

The proposed mechanism of SA on the electrode

Due to the mixed behavior of SA analysis on the chitosan@Fe_3O_4/CPE electrode, the adsorption between SA and amine group of chitosan was suggested as shown in Figure S2. As known SA is weak acid with pKa = 2.98 [23], SA in acidic medium (pH 5) formed salicylate ion giving negatively charge that can be adsorbed with amine in chitosan. This occurrence can confirm by giving higher sensitivity when the deposition time was increased as mentioned in S1(a). Thus, the proposed electrode gave the high sensitivity for SA analysis especially in pharmaceutical samples.

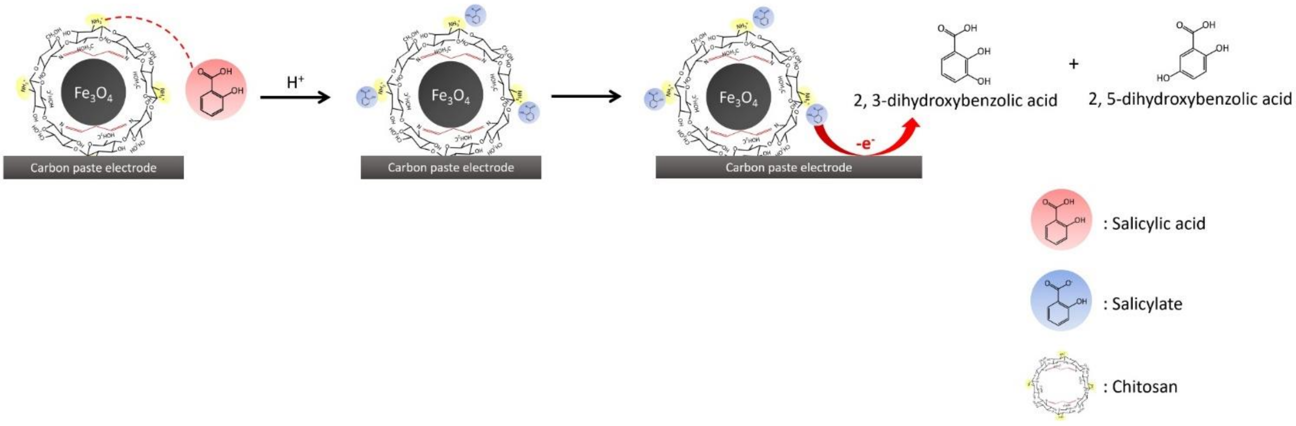

Figure S2. The proposed mechanism of SA on the chitosan@Fe_3O_4/CPE electrode.
